# Bronchial Anthracotic Change in South Khorasan Province (Iran), Emphasizing its Association with Tuberculosis

**Published:** 2014-09

**Authors:** Sayyed Gholamreza Mortazavi-Moghaddam, Sayyed Alireza Saadatjoo

**Affiliations:** 1 Department of Internal Medicine, Pulmonary Division, Vali-e-Asre Hospital, Birjand University of Medical Sciences, Birjand, Iran;; 2 Member of Diabetes Research Center, Faculty of Nursing and Midwifery, Birjand University of Medical Sciences, Birjand, Iran

**Keywords:** Anthracosis, Tuberculosis, Bronchial diseases

## Abstract

**Background: **There are many reports on the association between anthracosis and tuberculosis. This study focuses on bronchial anthracosis and associated diseases in the province of South Khorasan-Iran.

**Methods:** This case-series study is performed on patients referred to the Vali-e-Asre Hospital (South Khorasan-Iran) for bronchoscopic evaluations during the period of 2009-2012. Written informed consents were obtained prior to bronchoscopic evaluations. The criterion for diagnosis of bronchial anthracosis was black pigmentation on direct observation of bronchus. Bronchial anthracosis was classified into simple (without deformity) or complicated (with deformity). Pulmonary tuberculosis (TB) was diagnosed either by acid fast staining and culture of liquid samples, or histopathology examination of biopsy. Spirometry was performed to determine the obstructive or restrictive pattern.

**Results: **Among 279 patients who underwent bronchoscopic evaluations, 89 patients, including 34 males (38.2%) and 55 (61.79%) females, were diagnosed with anthracosis. Simple and complicated anthracosis were observed in 42 (48.2%) and 47 (52.8%) cases respectively. Mean age of patients was 72.23±9.65 years. There were 43 (48.3%) cases of tuberculosis (28 cases with complicated and 15 cases with simple anthracosis) (P=0.021). Chest X-ray showed consolidation/infiltration, reticular/fibrotic, and mass/nodule/hilar prominence in 57 (64%), 26 (29.21%) and 6 (6.74%) cases, respectively. Bronchitis was reported in 42 (%59.15) out of 79 patients whose biopsy samples were taken. Spirometric patterns were obstructive, restrictive, upper airway obstruction, and normal in 45 (50.56%), 32 (35.95%), 2 (2.24%), and 10 (11.23%) patients respectively.

**Conclusion: **Tuberculosis is the most frequent disease associated with anthracosis in South Khorasan province. Consequently, patients with anthracosis must be carefully evaluated for tuberculosis.

## Introduction

Bronchial anthracosis is defined as black discoloration of bronchus due to deposition of carbon particles from extraneous origin on bronchial mucosa.^1^ Bronchial anthracosis can be simple (without deformity) or complicated (with deformity and narrowing of bronchial lumen).^2^^,^^3^ Pulmonary disease due to inhalation of suspended particles in the air at work environment (including anthracosis) was first introduced by Ramazzini (the father of occupational lung diseases, 1703).^4^ From then on, occupational lung diseases became more and more attractive. Anthracosis as an independent lung disease was described for the first time in 1998.^5^

The main cause of anthracosis is unknown, however, prolonged contact with fossil fuel emissions has frequently been reported.^6^^,^^7^ When carbon particles remain in bronchial membrane for a prolonged time, it may lead to black discoloration, fibrotic changes and stenosis of bronchus.^8^^-^^11^ These particles are transported by the lymph to other organs.^12^

Carbon is the main component of biomasses and fossil fuel combustion being dispersed into the air. Considering the presence of carbon particles in the environment of urban/industrial areas and despite the fact that bronchoscopy is performed nearly in all referral medical centers, bronchial anthracosis is not widely reported. While there is no available report from Europe until 2008,^13^ the first report from Spain was in 2012.^14^ Reports from different parts of Iran are also not identical. For instance, while there are reports of the disease from Mashhad, Zahedan, Tabriz, Kerman, and Tehran, until recently there was no report of the disease from Farse province (Shiraz).^15^ Clearly, as in other areas, the residents of Farse province (Shiraz) are also exposed to fossil fuel emission.

This leads to the assumption that, in addition to fossil fuel emission, other factors such as genetics, chronic inflammatory reactions, balance of deposition and clearance of carbon particles, and/or chronic infections such as tuberculosis (TB) play a role in the development of bronchial anthracosis or anthracofibrosis.

Studies have shown that carbon deposition and clearance of particles is influenced by many factors. For instance, carbon deposition rate decreases in smokers due to increased mucus secretion and expectoration.^16^ While some researchers believe that tuberculosis is uncommon in anthracosis and anthracofibrotic lung diseases,^17^ others frequently report the coexistence of bronchial anthracosis with tuberculosis.^6^^,^^7^ Based on available data, tuberculosis is the most common associated disease among anthracosis cases and is reported to be between 30 to 60%.^15^^,^^18^ Bronchitis and airflow obstruction are also the most pathologic and functional abnormalities reported in cases with anthracosis.^19^^,^^20^ Regional studies on anthracosis and associated diseases would help to better understand the nature of the illness. Due to the lack of comprehensive report on the disease in South Khorasan, this study collects information on patients with anthracosis and associated diseases.

## Patients and Methods

The present investigation is a case-series study conducted from early 2009 until the end of 2012. Patients were selected among those referred to the Vali-e-Asre Hospital for bronchoscopic evaluations. These were selected mainly as a result of chest X-ray (CXR) abnormalities and/or undiagnosed respiratory problems. This hospital is the only referral center for bronchoscopic procedure in South Khorasan Province. Written informed consents were obtained prior to bronchoscopic procedures. Bronchoscopic procedures were performed by a pulmonologist under local anesthesia with oxygen saturation monitoring. Diagnostic criteria for bronchial anthracosis was based on direct observation of bronchial tree,^1^ thus, in view of bronchoscopy, patients with black discoloration of bronchus were selected and labeled as bronchial anthracosis. Bronchial anthracosis was classified as simple (without bronchial deformity) or complicated (with bronchial deformity and stenosis). In patients’ history, the use of wood and firewood for baking and cooking on traditional furnaces as well as exposure to fumes from contaminated fuel in kerosene heaters were recorded. Based on the needs and tolerance of patients, one of the bronchoscopic sampling methods (lung biopsy, bronchial biopsy; bronchial washing or bronchoalveolar lavage) was performed. Patients with mild mottled simple anthracotic change on the bronchi were excluded from bronchial biopsy*.* Bronchial and lung biopsies, bronchial washing or bronchoalveolar lavage samples were sent to pathology laboratory and examined by an expert pathologist regarding cytopathological and histopathological change. Considering the importance of TB, samples were also analyzed in two separate laboratories devoted to the study of tuberculosis. Samples were centrifuged and the sediments of samples were stained by the Ziehl Nelson method. The stained specimens were evaluated by two experts regarding tubercle bacillus. The sediments were also immediately inoculated onto Lowenstein-Jensen culture medium. The mycobacterium tuberculosis colonies were identified by colony characteristics. The presence of pulmonary tuberculosis was confirmed by either granuloma on histopathologic study of the obtained biopsy, positive acid fast smear or positive culture for tuberculosis. Causes of granuloma other than TB were excluded through more specialized investigations. Past history of tuberculosis was considered as old tuberculosis. Spirometry was performed using Cosmed Pony FX Desktop Spirometer (Italia). Upon laboratory confirmation on the presence of active tuberculosis, spirometry was performed two months after starting anti-tuberculosis therapy in order to prevent equipment contamination and transmission of tuberculosis. Spirometric parameters including FVC, FEV1, and FEV1/FVC were recorded according to the Global Initiative for Chronic Obstructive Lung Disease (GOLD). Patients were divided into restrictive group (normal or mildly reduced FEV1>80%, FVC<80% of predicted, and FEV1/FVC ratio>0.7) or obstructive group (FEV1<80% of predicted and FEV1/FVC ratio<0.7). Flow-volume spirometric loop was used to detect upper airway obstruction. Descriptive statistics and Chi-square test were used for data analysis. P value equal or less than 0.05 considered statistically significant.

## Results

From 279 patients who underwent bronchoscopy evaluation during early 2009 until the end of 2012, 89 (31.89%) patients, including 34 (%38.2%) males and 55 (61.79%) females were diagnosed with anthracosis.

The age range was from 45 to 105 years old. Mean age of the patients was 72.23±9.65 years and 58 out of 89 patients (65.16%) were 70 years old or over. Among patients with anthracosis, 36 (40.4%) were from urban and 53 (59.5%) were from rural areas. Exposure to fossil fuel emission (wood and firewood for baking and cooking on traditional furnaces or exposure to fume from contaminated fuel in kerosene heaters) was present in 79 cases (89%). Only 10 (11.23%) patients were smokers. Tuberculosis was diagnosed in 43 (48.3%) of cases (11 males and 32 females). Among patients with tuberculosis, 28 (65.11%) had active and 15 (34.88%) had old tuberculosis. Bronchial biopsy was taken in 71 out of 89 patients (79.77%) among which 42 (59.15%) cases were diagnosed with bronchitis. The symptoms and laboratory findings of these patients are summarized in table 1.

**Table 1:** Symptoms and laboratory findings of the studied cases with Anthracosis

**Findings**	**Number (%)**
Symptoms	
Dyspnea+cough (in both group of Anthracosis and Anthracofibrosis)	76 (85.39%)
Stridor+hoarseness (only in Anthracofibrosis)	9 (10.11%)
Hemoptesis(in both group of Anthracosis and Anthracofibrosis)	4 (4.49%)
Spirometry	
Obstructive	45 (50.56%)
Restrictive	32 (35.95%)
Normal	10 (11.23%)
Upper airway obstruction	2 (2.24%)
Pathologic reports (Biopsy report=71 cases)	
Bronchitis (non specific inflamation)	42 (59.15%)
Granuloma	14 (19.71%)
Fibrotic change	6 (8.45%)
Dysplasia	4 (5.63%)
Metaplasia	2 (2.81%)
Squamous cell carcinoma	1 (1.40%)
Hypersensitive pneumonitis	1 (1.40%)
Granulation tissue	1 (1.40%)
Radiological	
Consolidation/infiltration	57 (64.04%)
Reticular/fibrotic change	26 (29.21%)
Mass/nodule/hilar prominency	6 (6.74%)
Bronchus involvement	
Right side (main and lobar)	78 (87.64%)
Right main bronchus	46 (51.68%)
Right upper lobe	16 (17.97%)
Right middle lobe	8 (8.98%)
Right lower lobe	8 (8.98%)
Left side (main and lobar)	47 (52.8%)
Left main bronchus	38 (42.69%)
Left upper lobe	6 (6.74)
Left lower lobe	3 (3.37%)

There were 47 (52.8%) complicated and 42 (48.2%) simple anthracotic cases. Among 43 patients diagnosed with tuberculosis, 28 cases had complicated and 15 cases had simple anthracosis (P=0.021). Among 42 patients with bronchitis, 20 cases had complicated and 22 cases had simple anthracosis (P=0.23).

Consolidation/infiltration radiographic changes were observed in 57 (64%) out of 89 cases with anthracosis (table1). Tuberculosis was diagnosed in 32 (56.14%) cases with this type of radiographic changes among which 23 (82.14%) had active and 9 (60%) had old TB (P=0.013).

## Discussion

During the study period, among 279 patients that underwent bronchoscopy, 89 (31.89%) patients were diagnosed with anthracosis. Calculated percentage of anthracosis were 7%, 16.42%, and 20.86% for patient that undertook bronchoscopy at Amoli (Tehran 2004),^20^ Hemmati (Zahedan 2008)^21^ and Fekri (Kerman 2010)^22^ respectively. In comparison, the current study shows higher prevalence of the disease among patients undertaking bronchoscopy. However, based on the present study, true prevalence of the disease in South Khorasan province cannot be determined.

The age range was spread and the mean age of patients in the present study was 72.23±9.65 years, where 65% of them were over 70 years old. Virtually the same results could be seen in some other studies.^18^^,^^23^ It is likely that prolonged exposure to fossil fuel emission and chronic inflammatory response is necessary to establish the diseases.

More than 60% of patients in this study were females. Similarly, prevalence of the disease in females is higher than males in most studies.^18^^,^^22^ However, the opposite is also noticed in another study in Iran.^23^ Clarification for higher commonality of the disease among females could be due to their tendency to reside more at home and resort to traditional cooking.

In the present study, association between tuberculosis and anthracosis was observed in approximately 50% of cases. Some researchers believe that anthracosis may be a predisposing factor for tuberculosis.^24^ In contrast, some others believe that tuberculosis can lead to bronchial anthracosis alone without exposure to fossil fuel emission.^18^^,^^23^ There are reports from few regions where prevalence of tuberculosis among patients with anthracosis is ranged between 30 to 60 percent.^8^^,^^9^^,^^25^ Reported tuberculosis among patients with antracosis was 44% in Zahedan, 25-30% in Mashhad and 6.9% in Kerman.^16^ In contrast, there is no report of tuberculosis among patients with anthracosis from Tabriz and Sanandaj.^16^ It is worth mentioning that, prevalence of anthracosis is low among patients undergone bronchoscopy in Tabriz. During the study period, only 9 cases of anthracosis were reported from Tabriz.^16^ In areas such as Tehran, Mashhad, Kerman and Zahedan, prevalence of tuberculosis is relatively high and simultaneous anthracosis is frequently reported from these regions.^15^ However, such correlation is premature since until now, despite high prevalence of tuberculosis in areas such as Khuzestan, Golestan and Qom, anthracosis has not been reported.^26^

In the present study, tuberculosis was also more prevalent in patients with complicated anthracosis than those with simple anthracotic change. Therefore, it seems that anthracotic inflammation can induce more deformity when associated with TB.  Bronchial mucosal damage induced by tuberculosis or other inflammatory reactions can lead to fibrosis and bronchial stenosis.^8^ On this basis, it is believed that anthracofibrosis is actually a process of active tuberculosis.^8^ But such hypothesis is not confirmed in other studies.^19^ According to a study by Najafizadeh et al. there were no significant differences regarding the pattern of anthracotic change in patients with or without tuberculosis.^19^

Based on available evidence, there is a close relationship between anthracosis and tuberculosis.^18^ It is likely that these conditions jointly create synergistic effect on each other. While carbon particles are deposited in bronchial wall, continuous inflammatory phenomenon such as tuberculosis is required to trap carbon particles.

The patients in this study were not typical smokers and only 10 out of 89 patients were smokers. There was only one case of a 73-year-old male smoker with lung cancer. The result from the present study is similar to other reports indicating that lung cancer is rare among people with anthracosis.^23^^,^^27^ In spite of lung cancer being rare in patients with anthracosis, radiographic appearance mimicking lung cancer is common in these patients.^5^ Consequently all patients with anthracosis were carefully evaluated for malignancy, particularly among smokers.

In the present study, the most common histopathology finding on biopsy was bronchitis with nonspecific inflammation. Bronchitis is a distinct phenomenon but with multiple causes. Cigarette smoking is the most known cause but exposure to other fumes/dusts (including fossil fuel emissions) may also cause chronic bronchitis over a long period of time. Until recently, it was assumed that carbon particles suspended in the air are neutral in terms of disease induction. However, from one perspective, carbon with extraneous origin is the biggest worldwide environmental risk factor for lung’s health.^28^ Carbon particles are inhaled into bronchial airway and its subsequent chronic inflammation and fibrosis occurs.^10^ Bronchitis is an important complication of carbon particle inhalation. Some authors have applied terms such as; hut lung, charcoal disease, anthracotic inflammatory bronchial stenosis and antracobronchitis to distinguish this from other lung diseases caused by inhalation of other particles.^19^^,^^28^ Antracobronchitis can be an appropriate term for such patients when they are symptomatic. This term is also applied to this disease by other researchers.^19^ Considering the hypothesis that carbon particles are harmful to lung; bronchitis could be expected to be present in all patients in this study, although this has not been reported in the presence of a specific pathologic response such as granuloma.

Consolidation/infiltration involvement was the most common radiographic change observed on the CXR. Although not limited to patients with active tuberculosis, this type of abnormality was more common in patients with active tuberculosis. Consolidation/infiltration and other radiographic changes that can be observed in anthracosis and anthracofibrosis^20^ have not been approved to be specific for anthracosis or a particular associated condition in these patients.

In the current study, right-side involvement of bronchial tree was more common than the left-side. It is easier for particles to penetrate right bronchi and thus right-side bronchi are more frequently affected than left-side. There are also supporting evidences for this theory in other studies.^19^^,^^27^

Spirometry reveals airflow obstruction in most patients. Carbon particles derived from fossil fuel can be an important factor in causing bronchial airway disease.^19^^,^^20^^,^^29^^-^^32^

The present study had certain limitations. Due to ethical consideration, bronchoscopy could not be performed on healthy asymptomatic populations.  If such study was conducted, the actual prevalence of anthracosis and associated disease such as tuberculosis would have been obtained. In addition, severely ill patients were excluded due to their low tolerance level. It was not possible to specify bronchitis in anthracotic patients. Finally, because of ethical considerations, High Resolution Computerized Tomography (HRCT) of lung was not performed on all patients and HRCT changes were not included in the study.

## Conclusion

Observing TB in nearly 50% of the investigated patients re-emphasizes an interrelationship between TB and anthracosis. Given such observation, it is highly recommended that all patients with bronchial anthrachotic changes (particularly when associated with bronchial deformity) and with infiltration/consolidation changes on chest X-ray, to be meticulously evaluated in terms of TB. Furthermore, malignancy should be considered in smokers. Other associations with anthracosis are bronchitis and airflow obstruction. 
